# Endothelial ACKR1 is induced by neutrophil contact and down-regulated by secretion in extracellular vesicles

**DOI:** 10.3389/fimmu.2023.1181016

**Published:** 2023-04-21

**Authors:** Xinying Guo, Negar Khosraviani, Sneha Raju, Joshya Singh, Nikki Zamani Farahani, Madlene Abramian, Victor J. Torres, Kathryn L. Howe, Jason E. Fish, Andras Kapus, Warren L. Lee

**Affiliations:** ^1^ Keenan Centre for Biomedical Research, St. Michael’s Hospital, Toronto, ON, Canada; ^2^ Department of Laboratory Medicine and Pathobiology, University of Toronto, Toronto, ON, Canada; ^3^ Toronto General Hospital Research Institute, University Health Network, Toronto, ON, Canada; ^4^ Department of Microbiology, New York University Grossman School of Medicine, New York, NY, United States; ^5^ Department of Biochemistry, University of Toronto, Toronto, ON, Canada; ^6^ Department of Medicine and the Interdepartmental Division of Critical Care Medicine, University of Toronto, Toronto, ON, Canada

**Keywords:** atypical chemokine receptor 1, Duffy antigen, endothelium, neutrophil, extracellular vesicles, leukocidin

## Abstract

Atypical chemokine receptor-1 (ACKR1), previously known as the Duffy antigen receptor for chemokines, is a widely conserved cell surface protein that is expressed on erythrocytes and the endothelium of post-capillary venules. In addition to being the receptor for the parasite causing malaria, ACKR1 has been postulated to regulate innate immunity by displaying and trafficking chemokines. Intriguingly, a common mutation in its promoter leads to loss of the erythrocyte protein but leaves endothelial expression unaffected. Study of endothelial ACKR1 has been limited by the rapid down-regulation of both transcript and protein when endothelial cells are extracted and cultured from tissue. Thus, to date the study of endothelial ACKR1 has been limited to heterologous over-expression models or the use of transgenic mice. Here we report that exposure to whole blood induces ACKR1 mRNA and protein expression in cultured primary human lung microvascular endothelial cells. We found that contact with neutrophils is required for this effect. We show that NF-κB regulates ACKR1 expression and that upon removal of blood, the protein is rapidly secreted by extracellular vesicles. Finally, we confirm that endogenous ACKR1 does not signal upon stimulation with IL-8 or CXCL1. Our observations define a simple method for inducing endogenous endothelial ACKR1 protein that will facilitate further functional studies.

## Introduction

Atypical chemokine receptor-1 (ACKR1), also known as the Duffy antigen receptor for chemokines (DARC), is a blood group antigen that is expressed on erythrocytes and endothelial cells but not on leukocytes. While recognized as a receptor on erythrocytes for the parasite that causes malaria (1), its function on the endothelium is still not well understood.

Despite homology to other chemokine receptors and the ability to bind both CC and CXC chemokines, ACKR1 lacks the intracellular DRY (Asp/Arg/Tyr) motif present on most G-protein-coupled receptors that is required for signalling (2). Accordingly, ACKR1 expressed on erythrocytes has been postulated to act as a “sink” or reservoir, serving to modulate the inflammatory response by binding and releasing circulating chemokines. Intriguingly, a homozygous mutation in a GATA motif in the promoter region causes loss of ACKR1 expression on erythrocytes and confers protection from malaria but leaves endothelial ACKR1 expression unaffected (3, 4). This suggests that ACKR1 is subject to tissue-specific regulation and may also reflect an important function in the vasculature. Studies from human and murine tissues have indicated that endothelial ACKR1 is restricted to post-capillary venules (5, 6) but there is relatively little literature on its regulation or function. The paucity of studies is attributable in part to the fact that ACKR1 expression disappears in cultured endothelial cells within hours of isolation from tissue (7), complicating *in vitro* experiments. Endothelial cells in culture display phenotypic instability, for instance rapidly losing caveolae once isolated from tissue (8) and demonstrating marked changes in gene expression; mRNA for ACKR1 and the protein disappear within hours (7). *In vitro*, the cytokine TNFα has been reported to induce ACKR1 mRNA (9) but only modestly increased protein levels by human umbilical vein endothelial cells; stimulation of the cells with other cytokines (IL-1, IL-8) and growth factors (e.g. vascular endothelial growth factor (VEGF), platelet-derived growth factor) had no effect on ACKR1 expression.

As a result, almost all studies on endothelial ACKR1 have required either *in vivo* approaches or heterologous over-expression. The ability to study endogenous ACKR1 on cultured endothelial cells might facilitate our understanding of its regulation and function. We recently discovered that incubation with human whole blood causes induction of endogenous ACKR1 on cultured primary human microvascular lung endothelial cells (10); this in turn conferred susceptibility to a pore-forming leukocidin secreted by *S. aureus* which binds plasmalemmal ACKR1 and causes cell death. However, how blood induces endothelial ACKR1 remains unknown.

Here we report that whole blood induces endothelial ACKR1 mRNA transcription and protein expression; this effect was recapitulated by physical contact between neutrophils and the endothelium. Endothelial NF-κB mediates ACKR1 induction and ACKR1 expression is rapidly down-regulated through its secretion in extracellular vesicles. Consistent with studies of the heterologously expressed protein (11), endogenous ACKR1 on the endothelium did not signal upon stimulation with IL-8 or CXCL1.

## Methods

### Cell culture

Primary human pulmonary microvascular endothelial cells (HPMECs, PromoCell: C-12281) were cultured in Lonza Endothelial Cell Basal Medium-2 (EBM-2, CC-3156), supplemented with Lonza EGM-2MV (CC-4147) without gentamicin and grown in 37°C with 5% CO_2_. The medium was changed every 2–3 days of culture. All experiments were performed on cells between passage 5-7.

### Blood collection

The Research Ethics Board (REB) of St. Michael’s Hospital, Toronto, Canada, approved the use of healthy human volunteer blood for induction of ACKR1 *in vitro* under protocol REB#17-184. We are not given permission to collect identifying information or personal health information in order to protect the confidentiality of the volunteers. Human whole blood from healthy individuals was collected in heparin sodium-coated vacutainers for *in vitro* experiments.

### Isolation of blood components

For plasma isolation, blood was centrifuged at 2500 rpm for 15 minutes at room temperature and the plasma was collected and incubated with confluent HPMECs. For isolation of cellular components, whole blood was first diluted with an equal volume of phosphate buffered saline (PBS). Diluted blood was loaded on top of the Mono-Poly™ Resolving Medium (MP Biomedicals 091698049) and centrifuged at 800g for 45 minutes at room temperature. Mononuclear and polymorphonuclear leukocytes from whole human blood were separated in two distinct bands and erythrocytes were at the bottom of the tube. After centrifugation, isolated cellular components were collected, washed with PBS twice and resuspended in complete media. Isolated cells were then incubated with HPMECs to attempt induction of ACKR1.

### ACKR1 induction

HPMECs were grown to confluency on 6 well plates and incubated with 1ml of whole blood or the same volume of complete media as control for 24 hours. For the isolated cellular components, HPMECs were incubated with resuspended monocytes, polymorphonuclear leukocytes and erythrocytes respectively for 6 hours. Following incubation, cells were washed with PBS and processed for immunoblotting or qPCR.

### Inhibitor treatments

To attempt to inhibit the loss of ACKR1, HPMECs were first incubated with whole blood for 24 hours for ACKR1 induction. Cells were then washed with PBS and treated with MG-132 (20μM; CAS 133407-82-6), Marimastat (100μM; Santa Cruz, sc-202223), Bafilomycin A (100nM; EMD Millipore Cat# 5.08409.0001), GW4869 (2μM; Millipore Sigma CAS 6823-69-4) or DMSO in complete media for 6 hours. Cells were then lysed and processed for immunoblotting. For inhibition of protein synthesis, cells were treated with cycloheximide (100μg/ml; Sigma 01810) at the time of incubation with isolated PMNs for 6 hours. For inhibition of NF-κB signaling, HPMECs were pretreated with Bay 11-7082 (20μM; Sigma, B5556) for 30 minutes in complete media and then inhibitors were washed out and replaced with blood for 6 hours for ACKR1 induction.

### Immunofluorescence

HPMECs were fixed with 4% paraformaldehyde (PFA) at room temperature for 15 minutes. After washing twice with PBS, any remaining PFA was inactivated with 100 mM glycine for 20 minutes. Cells were then permeabilized with 0.1% Triton-X in 1% BSA-PBS for 20 minutes. After permeabilization, HPMECs were blocked with 5% goat serum for 1 hour and then incubated with primary antibody against ACKR1 (Novus NBP2-75197) and CD31 (sc-376764, Santa Cruz Biotechnology) at a concentration of 10 μg/ml and 4 μg/ml respectively (1 hour at room temperature). HPMECs were washed five times with PBS and incubated with their corresponding Alexa Fluor-conjugated secondary antibodies (Jackson ImmunoResearch, 115-546-006, 111-605-003) at 1.5 μg/ml in PBS. Cells were washed 5 times with PBS and mounted in mounting media supplemented with 1 μg/ml DAPI. Images were acquired with a spinning disk microscope (Olympus IX81, at 63x objective) at a z-stack interval of 0.3 μm; all settings were kept constant between groups in the same experiment. Live cell microscopy was used to assess endosomal acidification after treatment with bafilomycin A. HPMECs seeded on 25mm glass coverslips were treated with or without bafilomycin A at 100 nM for 1 hour in complete media. After washing 3 times with PBS, coverslips were treated with 1 μM cresyl violet and NucBlue in HBSS and incubated for 5 minutes before imaging in a live cell imaging chamber on the spinning disk microscope. Images were acquired with a z-stack interval of 0.3 μm and settings were kept constant between groups.

### Immunoblotting

Before collecting cells for immunoblotting, HPMECs were washed at least 5 times with cold PBS to remove any blood cells. Protein lysate was prepared in SDS lysis buffer (62.5 mM Tris-HCl pH 6.8, 2% SDS, 10% glycerol) and denatured in 5x loading buffer at 95°C for 10 minutes. Bradford protein assay was performed and 50 μg of protein was run on an SDS-PAGE using 10% polyacrylamide gel, running at 150V. Proteins were transferred onto PVDF membrane and transferred for 70 minutes at 110V. Membranes were washed three times with TBS-0.1% Tween-20 (TBS-T) and blocked with 5% BSA in TBS-T. Membranes were then washed and incubated with 1:1000 primary antibodies overnight at 4°C: anti-α-actinin (rabbit, CST #31345), anti-ACKR1 (rabbit, abcam ab58965& rabbit, Novus NBP2-75197), anti-ubiquitin (P4D1; mouse, CST#3936), and anti-β-actin (C4; mouse, Santa Cruz sc-47778), anti-PECAM-1(h-3) sc-376764, anti-ICAM-1(15.2) sc-107, anti-p-p44/42(T202/y204) CST#9101, anti-p44/42 Erk1/2 CST#9102, anti-pAkt (S473) CST#9271, anti-Akt (pan40D4) CST#2920, anti-p-p38MAPK (T180/Y182) CST#9215, anti-p38 CST#9212, anti-p105/p50(D4P4D) CST#13586, and anti-LC3B CST#2775. The following day, membranes were washed three times with TBS-T and incubated in 1:10000 secondary antibody in TBS-T for 1 hour at room temperature: anti-mouse IgG HPRT (CST#70763) and anti-rabbit IgG HPRT (CST#7074). Membranes were washed and visualized using enhanced chemiluminescence, imaged on the ChemiDoc™ Imaging System (Bio-Rad), and quantified using ImageLab software (Bio-Rad).

### RNA extraction and quantitative real time PCR

RNA was extracted using the Trizol-chloroform method and the Qiazol lysis reagent (Qiagen, 79306). cDNA was synthesized using the High-Capacity cDNA Reverse Transcription kit according to the manufacturer’s protocol and was performed on the Eppendorf Mastercycler gradient thermal cycler. qPCR was conducted using the PowerUp™ SYBR® Green Master Mix (Applied Biosystems) and it was performed on the QuantStudio™ 7 Real-Time PCR System (Applied Biosystems) where the cDNA was denatured at 95°C for 10 minutes followed by 40 cycles of 95°C for 15 seconds then 60°C for 1 minute. Data was analyzed using primers for the following genes: GAPDH (Forward: 5’-CAA TGA CCC CTT CAT TGA CC-3’, Reverse: 5’-GAC AAG CTT CCC GTT CTC AG-3’); ACKR1 (Forward: 5’-GTC TTG TTG CCA TTG GGT TT-3’, Reverse: 5’-GAC AAC AGC AAC AGC TTG GA-3’).

### Transwell experiment

HPMECs were seeded (120 000 cells/well) onto a 12-well plate and grown until a confluent monolayer was obtained. Cells were incubated with either complete media, blood or isolated PMNs added to the top chamber of 0.4 µm Polyester Membrane Transwells (Costar, REF 3460). Cells were incubated for 24 hours for whole blood induction and 6 hours for PMN induction then lysed and processed for western blot and qPCR.

### Chromatin immunoprecipitation

Cells were grown in 10 cm dishes and (once confluent) were incubated with blood or complete media for 24 hours. Cells were then fixed using 1% formaldehyde for 10 minutes, followed by 125 mM glycine for 5minutes. Cells were washed with cold PBS and lysed using a whole cell lysis buffer (5 mM PIPES, 85 mM KCl, 0.5% NP40) then incubated on ice for 10 minutes and centrifuged at 1000 rpm for 10 minutes at 4°C. The supernatant was discarded, and the pellet was resuspended in nuclei lysis buffer (50 mM Tris-HCl, 10 mM EDTA). Samples were sonicated using Covaris M220 Focused Ultrasonicator to achieve fragment size of 200-400 bp. 20% input was set aside, and the samples were incubated with 2.5 μg of anti-RNA polymerase II (8WG16) (Santa Cruz, sc-56767) or normal mouse IgG (Millipore Sigma, 12-371) as control diluted in buffer (1:10) and incubated overnight at 4°C. Samples were incubated with Protein G Dynabeads for 2 hours at 4°C. Beads were washed (low salt buffer, high salt buffer, LiCl buffer, then TE buffer) and DNA-protein complex eluted using elution buffer (1% SDS, 100 mM NaHCO_3_). DNA was purified using Geneaid Gel/PCR DNA extraction kit followed by RT-qPCR.

### Calcium mobilization assay

HPMECs were seeded onto 0.1% gelatin-coated 25 mm glass coverslips at a density of 500 000 cells/ml. Once confluent, cells were incubated with human whole blood or complete media for 24 hours. Cells were washed with Hank’s Buffer Saline Solution (HBSS) without Ca^2+^ and loaded with Fura2-AM (10 ng/μl) in HBSS for 30 minutes at 37°C. Cells were washed three times with HBSS and incubated in HBSS for 15 minutes. Coverslips were transferred to a cell chamber and fluorescence of Fura2-AM was measured using ratiometric microscopy. Cells were treated with100 ng/ml of IL-8, 40 ng/ml VEGF, 5μM calcium ionophore, and 1mM MnCl_2_. Fluorescence was measured every second and calcium concentration was calculated using Felix software. Baseline calcium concentration was measured for 5 minutes, followed by 10 minutes of IL-8 treatment. For VEGF, fluorescence was measured until a peak in 340 nm/380 nm ratio was observed followed by a drop to the baseline. Once measurements were made, cells were lysed and processed for immunoblotting for ACKR1 as above. The maximum and minimum ratio of 340 nm/380 nm were obtained from the ionophore and MnCl_2_ respectively and used in the equation below:


$$\left[Ca^{2+}\right]=Kd\frac{R-v\times Rmin}{v\times Rmax-R}\times \frac{Sf2}{Sb2}$$

where R is 340 nm/380 nm ratio, Kd = 224, Sf2 and Sb2 is 1, and v (viscosity) is 1. This calculates the calcium concentration for each time point measured. The average of the first 10 highest concentrations at baseline, IL-8 and VEGF were made and normalized to baseline for graphical presentation.

### siRNA transfection

Depletion of ICAM-1 and p50 was accomplished by transfection of siRNA with Lipofectamine™ RNAiMAX Transfection Reagent (Invitrogen) as per the manufacturer’s protocol. Cells were transfected with 20nM of target siRNA or negative siRNA as control. ACKR1 induction by whole blood was performed 48 hours post transfection. All siRNAs were purchased from Qiagen (Valencia, CA, USA): the negative targeting control (NTC) siRNA (Cat. No. 1027310), ICAM-1 (Cat. No. GS3383), and p50 (Cat. No. GS4790).

### Extracellular vesicle isolation 

HPMECs were grown to confluency in 15cm dishes, and incubated with blood or complete media for 24 hours. After blood exposure, blood cells were removed by washing with PBS followed by serum-free media. EVs were isolated from the supernatant collected 6 hours post blood (or media) removal. After 500*g* and 3000*g* spins to remove apoptotic cells and cell debris, supernatants were filtered using a 0.22 µm syringe filter to enrich predominantly exosomes and concentrated to 500 µl using Amicon 10K MWCO filters (12, 13). EVs were enriched using qEV legacy size exclusion chromatography columns (Izon Science Ltd, Christchurch, NZ, SP1) as per the manufacturer’s instructions. Briefly, 500 µl of concentred cell culture supernatant was loaded onto the qEV columns followed by elution with PBS^-/-^. After collection of a three-millilitre void volume, four fractions of 500µl volumes were collected from the column. The EV enriched fractions were concentrated to 30 µl via ultrafiltration using the Amicon 10K MWCO filters (Millipore, UFC801096D) before subsequent experiments.

### Western blotting for EV markers

EV samples were lysed in RIPA buffer (CST#9806) prior to protein quantification via Micro BCA (ThermoFisher, 23235) and gel electrophoresis. Protein quantification was completed as per the manufacturer’s protocol with 2-3 µL of lysed EV sample. Laemmli buffer was added followed by 2.5% β-mercaptoethanol prior to boiling at 70°C for 10 minutes for CD63 or 95°C for 5 minutes for all other markers. Proteins were separated on 4-20% precast polyacrylamide gels (Mini-Protean TGX Precast Protein gels, 4561094) for 30 minutes at 50V followed by 105 minutes at 70V. Gels were transferred to activated PVDF membranes for 90 minutes at 50V. Membranes were blocked for 1 hour in 5% milk in TBS-T and incubated with primary antibodies in 5% milk overnight. Primary antibodies utilized were anti-CD63 (ABclonal A5271, 1:1000) and anti-CD81 (ABclonal A5270, 1:1000). Membranes were then incubated with anti-rabbit HRP (Cell Signalling, 7074S) in 5% milk in TBS-T for 1 hour at room temperature. Membranes were developed using SuperSignal West Femto Maximum Sensitivity Substrate ECL (ThermoFisher, 34094) and imaged on Bio-Rad ChemiDoc system.

### Exposure of HPMEC cells to leukocidin after induction of ACKR1

The leukocidin HlgAB was purified from *S. aureus* culture supernatants as described previously (10). Purified HlgAB was tested for LPS with the ToxinSensor Chromogenic LAL Endotoxin Assay Kit (GenScript) and found to contain less than 0.006 EU/mL. HPMEC cells were grown and treated with isolated PMN for 6 hours or whole blood for 24 hours as above. The cells were incubated with 0.4 μM of HlgAB in serum-free EGM-2 media for 1 hour. Propidium iodide (PI, 10μg/ml) and Nucblue were then added to the media. The fraction of the total cells positive for PI was determined using the BioTek Cytation5 microscope and Gen5 software.

### Statistics

Statistical analysis was performed using GraphPad Prism software (GraphPad Prism 9.0.0; GraphPad Software Inc., La Jolla, CA, USA). Unpaired *t*-tests, ordinary one-way ANOVA with Dunnett’s multiple comparisons test, and ordinary two-way ANOVA with Šídák’s multiple comparisons test (GraphPad, La Jolla, CA, USA) were used to determine the significance of raw or normalized data (respectively). Data are presented as mean ± SEM. A p-value of <0.05 was considered significant. All experiments were performed at least three times independently on different batches of cells.

## Results

### Exposure to whole blood induces ACKR1 protein and mRNA expression in primary human lung microvascular endothelial cells

**Figure 1:**
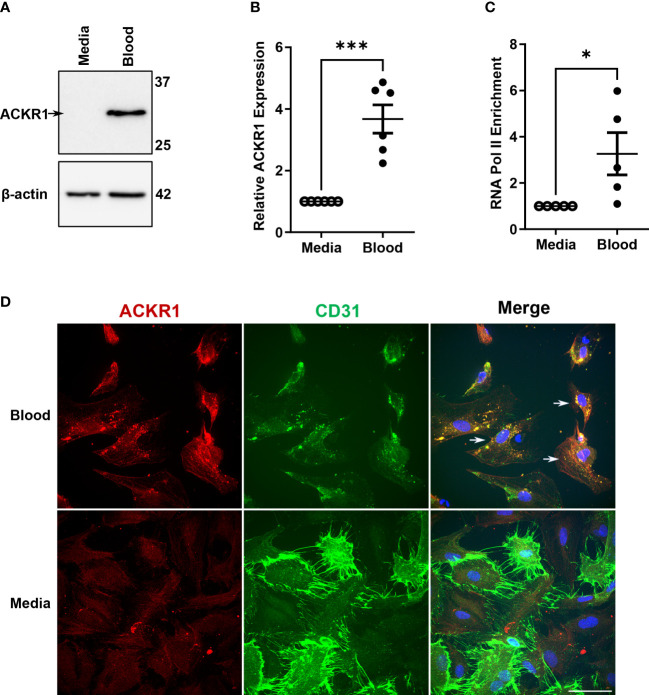
Incubation with blood induces ACKR1 mRNA and protein in primary human lung microvascular endothelial cells. **(A)** Immunoblotting for ACKR1 using the Ab58965 antibody in cells incubated with blood or complete media for 24 hours. **(B)** qPCR for *ACKR1* mRNA under the same conditions (***p<0.001). **(C)** Chromatin immunoprecipitation assay using antibody to RNA polymerase II or IgG control and primers for the *ACKR1* coding region after treatment of cells as in A) (*p<0.05). **(D)** Immunostaining of endothelial cells for ACKR1 (red) after 6 hours incubation with blood; the endothelial membrane protein CD31 is stained in green. Note increased ACKR1 expression in the blood-exposed cells that are positive for CD31 (white arrows in merged image).

We reasoned that the disappearance of *ACKR1* mRNA and protein from endothelial cells isolated from tissue was due to the loss of cues from the *in vivo* environment. Accordingly, we exposed primary human lung microvascular endothelial cells (HPMECs) to human whole blood for 24 hours. As we have previously reported (10), this exposure was sufficient to increase ACKR1 protein levels on cultured HPMECs (**Figure 1A**) and was consistent across different blood donors and different antibodies (**Supplemental Figure 1**). Since ACKR1 is also known to be highly expressed on the erythrocyte surface and to exclude its transfer to the endothelium [e.g. by trogocytosis (14)], we measured mRNA and found that exposure to blood significantly induced *ACKR1* mRNA levels in endothelial cells (**Figure 1B**); baseline levels of this transcript are very low (e.g. cycle count 28-32). To confirm that gene transcription of *ACKR1* was induced in the endothelium, we then performed chromatin immunoprecipitation (ChIP) for RNA polymerase II (RNA Pol-II) followed by PCR using primers for the *ACKR1* coding region. We observed a significant increase in RNA pol-II enrichment at the *ACKR1* gene in endothelial cells incubated with blood compared to controls. As erythrocytes are anucleate, this indicates that *bona fide* endothelial *ACKR1* transcription was induced (**Figure 1C**). Finally, we examined the subcellular localization of endothelial ACKR1by immunofluorescence after the cells were exposed to blood. Immunofluorescence confirmed that ACKR1 was expressed on endothelial cells as indicated by staining for CD31, an endothelial membrane protein (**Figure 1D**). Together, these experiments indicate that exposure to blood induces *de novo* protein synthesis of ACKR1 in cultured primary lung microvascular endothelial cells.

### Induction of ACKR1 by whole blood requires endothelial contact with neutrophils

**Figure 2:**
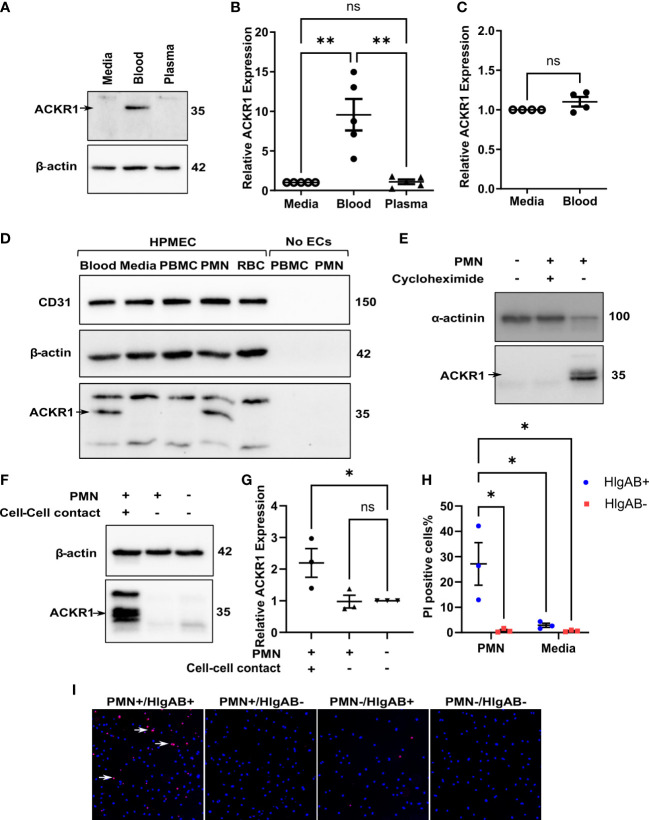
Induction of endothelial ACKR1 by blood is recapitulated by physical contact with neutrophils. **(A)** Immunoblotting for ACKR1 in cells incubated with media, blood, or plasma for 24 hours. **(B)** qPCR for *ACKR1* mRNA under the same conditions (**p<0.01). **(C)** qPCR for *ACKR1* mRNA from endothelial cells seeded in the lower chamber of a 0.4 μm-pore transwell with whole blood added to the upper chamber for 24 hours (ns, not statistically significant). **(D)** Fractionation of the cellular components of human blood by density centrifugation followed by incubation with human lung microvascular endothelial cells (HPMEC) for 6 hours; lysates were probed for ACKR1 by immunoblotting using the NBP2-75197 antibody. As a control, peripheral blood mononuclear cells (PBMC) and polymorphonuclear leukocytes (PMN; neutrophils) were also probed for ACKR1 in the absence of endothelial cells. **(E)** Lung microvascular endothelial cells were incubated with human neutrophils in the presence or absence of cycloheximide for 6 hours; this was followed by immunoblotting for ACKR1. **(F)** Incubation of lung endothelial cells with human neutrophils either directly (cell-cell contact) or with separation by a transwell as in B) for 6 hours followed by immunoblotting for ACKR1. **(G)** qPCR for *ACKR1* mRNA under the same conditions (*p<0.05). **(H)** After exposure to PMN for 6 hours, lung microvascular endothelial cells were treated with the leukocidin HlgAB (0.4 μM) for 1 hour, followed by propidium iodide (PI) and NucBlue staining. The percentage of PI-positive cells is shown; *p<0.05. **(I)** Representative images of PI uptake (red) by cells after HlgAB treatment.

We next wished to determine which cellular or soluble constituent of blood was responsible for the induction of endothelial ACKR1. Incubation with isolated plasma failed to induce either ACKR1 protein or mRNA (**Figures 2A, B**). In addition, physical separation of the blood from the endothelial cells using 0.4 μm-pore transwells prevented the induction of ACKR1 (**Figure 2C**). These observations suggested that physical contact with a cellular component of blood was likely required. We fractionated blood by density centrifugation, isolating peripheral mononuclear cells (PBMC), polymorphonuclear leukocytes (PMN, neutrophils) and erythrocytes (RBC). We exposed lung microvascular endothelial cells to these purified fractions and observed that only the PMN fraction was capable of recapitulating the induction of ACKR1; isolated PMN were devoid of the protein (**Figure 2D**). Using cycloheximide and transwells respectively, we observed that ACKR1 induction by PMN required both protein synthesis (**Figure 2E**) and physical contact between PMN and endothelial cells (**Figures 2F, G**). To confirm that ACKR1 induced by neutrophils recapitulated the effect of whole blood and was expressed on the cell surface (10), we exposed endothelial cells to the pore-forming leukocidin HlgAB and measured cell death by uptake of propidium iodide. Only cells that had been incubated with neutrophils were susceptible to the toxin (**Figures 2H, I**).

### Induction of ACKR1 by whole blood requires NF-κB but not ICAM-1 or endothelial nitric oxide synthase

**Figure 3:**
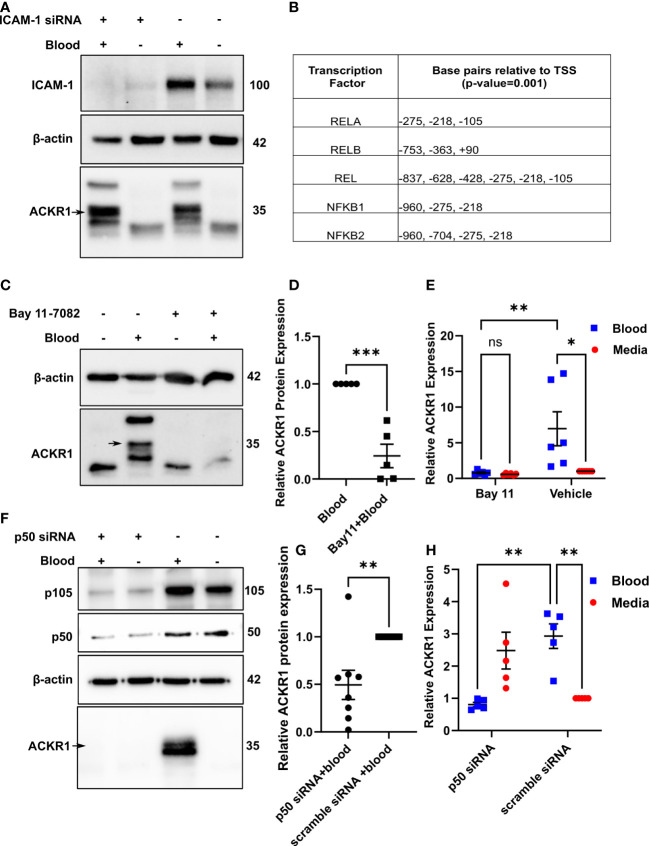
Neutrophils induce endothelial ACKR1 through NF-κB. **(A)** Lung endothelial cells were depleted of ICAM-1 by siRNA followed by incubation with blood for 6 hours. Immunoblotting of whole cell lysates for ACKR1 using the NBP2-75197 antibody was performed. **(B)** Table shows selected predicted promoter binding sites for *ACKR1*. **(C, D)** Endothelial cells were pre-treated with the NF-κB inhibitor Bay 11-7082 for 30 minutes followed by incubation with blood for 6 hours. Lysates were probed for ACKR1 (representative blot is shown); quantification in **(D)** (***p<0.001). **(E)** qPCR for *ACKR1* mRNA under the same conditions (**p<0.01, *p<0.05). **(F–H)** Endothelial cells were depleted of p50 by siRNA followed by incubation with blood for 6 hours; ACKR1 protein **(F)** and quantification in **(G)**; **p<0.01) and mRNA **(H)**; **p<0.01) are shown.

The induction of ACKR1 by neutrophil contact suggested that engagement of endothelial cell-surface proteins might be a trigger. In the lung microvasculature, ICAM-1 is critically important to neutrophil adhesion and subsequent emigration (15). Accordingly, we depleted the protein by siRNA before exposure to blood. Essentially complete depletion of ICAM-1 had no effect on induction of ACKR1 protein by blood (**Figure 3A**).

Using the EPD website, we next analyzed the 5’ region of the *ACKR1* gene for potential transcription factor binding sites. We observed several potential NF-κB binding sites within -1000 bp to +100 bp relative to the transcription start site with a cut-off (p-value) of 0.001 (**Figure 3B**). To test whether ACKR1 induction was regulated by NF-κB, we pretreated lung microvascular endothelial cells with the NF-κB inhibitor Bay 11-7082 (16) for 30 minutes before blood exposure. This significantly attenuated the effect of blood on *ACKR1* mRNA and protein expression (**Figure 3C–E**).To confirm these findings by an alternative and more specific method, we depleted p50 with siRNA and achieved partial knockdown. Partial depletion of p50 was sufficient to blunt the induction of ACKR1 protein and mRNA by blood (**Figures 3F–H**). Interestingly, cells depleted of p50 in the absence of blood showed a slight increase in mRNA for *ACKR1* but not protein. This may reflect basal repression of ACKR1 transcription by the p50 homodimer (17) even though p50/p65 heterodimers are most likely to drive activation of expression. Together, these data indicate that NF-κB is required for blood-induced ACKR1 expression.

### ACKR1 protein and mRNA levels diminish significantly within hours of blood removal

**Figure 4:**
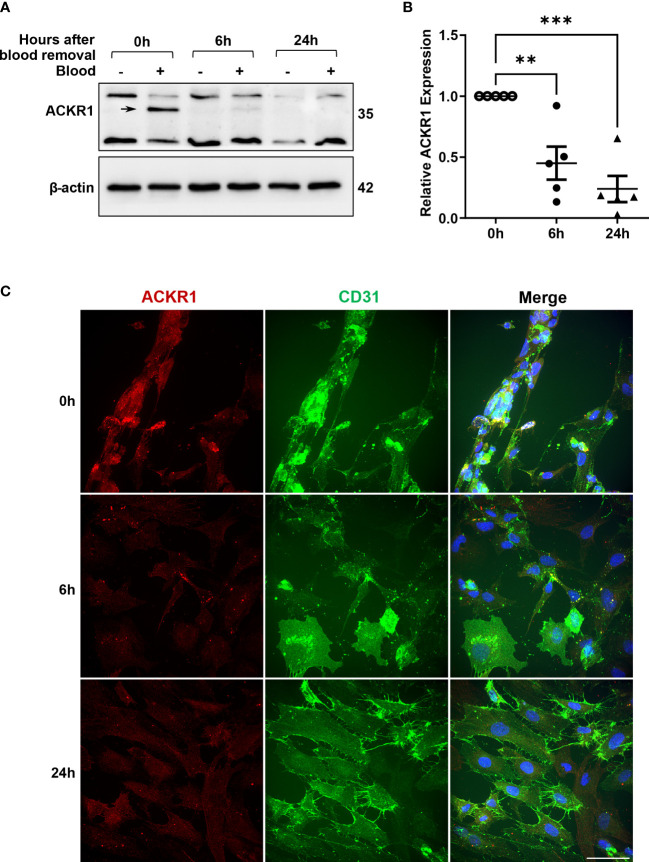
Rapid loss of ACKR1 protein and mRNA upon removal of blood. **(A)** After incubation with blood for 24 hours, blood was removed and endothelial lysates were probed for ACKR1 by immunoblotting using the Ab58965 antibody. **(B)** qPCR for *ACKR1* mRNA under the same conditions (**p<0.01; ***p<0.001). **(C)** Immunostaining for ACKR1 and CD31 under the same conditions.

**Figure 5:**
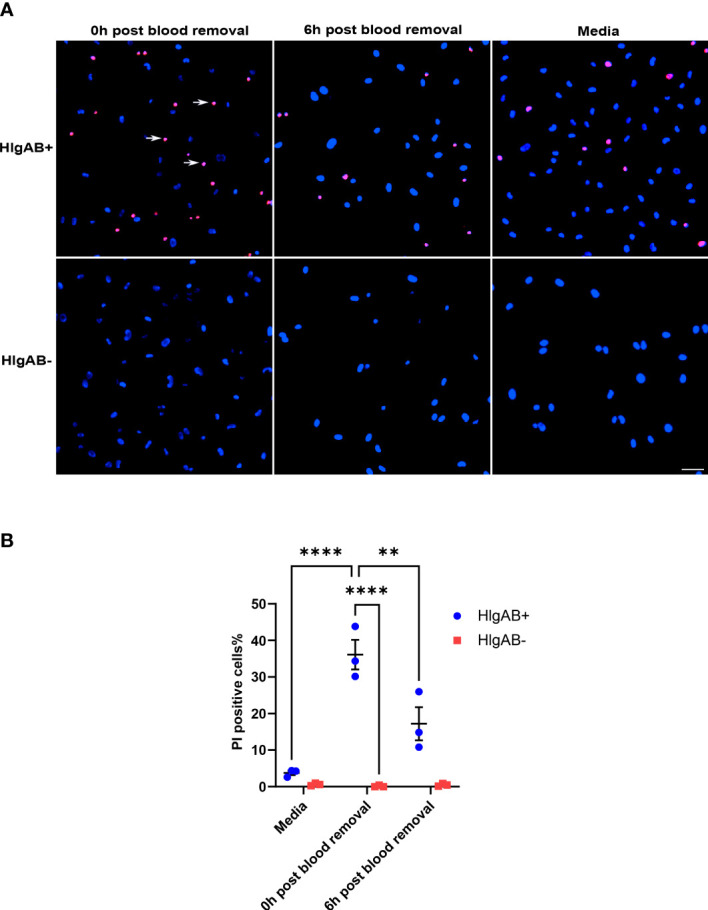
Susceptibility to the leukocidin HlgAB decreases as ACKR1 is cleared upon blood removal. **(A)** HPMECs were pre-incubated with blood for 24 hours. Either immediately after or 6 hours after blood was removed, cells were exposed to HlgAB for 1h. Apoptotic endothelial cells were detected by propidium iodide (PI) uptake (see white arrow in representative images). **(B)** Quantification of PI-positive cells among total cells (**p<0.01; ****p<0.0001).

Given that ACKR1 expression declines rapidly at both the protein and transcript level after endothelial cells are isolated from tissues, we wanted to determine whether its expression persists once blood is removed. Following incubation with blood for 24 hours, cells were rinsed and then incubated in complete media. We observed a significant decrease in protein levels within 6 hours of removal of blood with complete loss of ACKR1 after 24 hours (**Figure 4A**). Concordantly we measured mRNA levels and observed a significant decrease in *ACKR1* transcript over time (**Figure 4B**). We also performed immunofluorescent staining for ACKR1 at different time points after blood removal and confirmed its rapid loss in cultured lung microvascular endothelial cells (**Figure 4C**). Thus, both mRNA and protein for ACKR1 are rapidly cleared upon removal of blood. As an additional control for the loss of ACKR1, we measured the susceptibility of endothelial cells to the leukocidin HlgAB over time. While endothelial cells were killed by the toxin upon induction of ACKR1, their susceptibility diminished significantly 6 hours later coinciding with loss of the protein (**Figures 5A, B**).

### Loss of ACKR1 protein after removal of blood is not due to proteasomal, metalloproteinase, or lysosomal degradation

**Figure 6:**
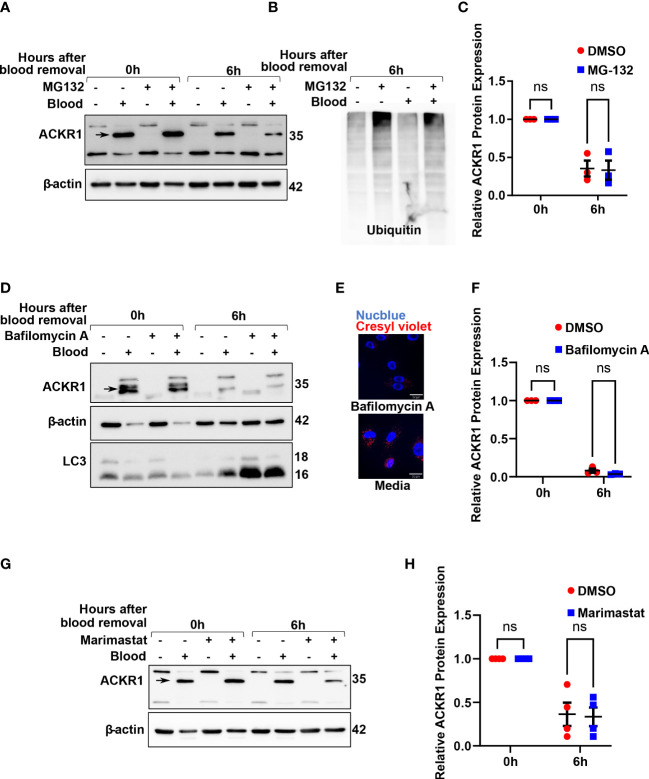
Inhibition of the proteasome, lysosomes/autophagy and metalloproteases do not prevent loss of ACKR1 from endothelial cells. **(A, B)** HPMECs were pre-incubated with blood for 24 hours and subsequently washed and incubated with media containing the proteasome inhibitor MG132 at 20 μM for 6 hours. Immunoblotting was used to assess ACKR1 protein (using the Ab58965 antibody) and ubiquitin levels. **(C)** Quantification of ACKR1 protein, normalized to β-actin. **(D)** Lysosomes and autophagy were inhibited with Bafilomycin A (100 nM) for 6 hours after blood removal. Immunoblotting was used to assess ACKR1 protein; LC3 is a control for autophagy. **(E)** Lysosomes were stained with Cresyl violet in live cells with or without Bafilomycin A treatment. **(F)** Quantification of ACKR1 protein, normalized to β-actin. **(G, H)** Cells were treated with Marimastat (100 μM) after blood removal to inhibit matrix metalloproteases; ACKR1 protein level was normalized to β-actin. ns means not significant.

To understand how ACKR1 is cleared from endothelial cells, we first considered the major protein degradation pathways: the ubiquitin-proteasome pathway and the lysosomal proteolysis/autophagic degradation pathway (18). Inhibition of the proteasome using MG-132 (19) had no effect on ACKR1 protein levels upon blood removal (**Figures 6A–C**); this is despite obvious accumulation of ubiquitinated proteins in cell lysates indicating successful proteasomal inhibition. Next, we inhibited lysosomal degradation using the vacuolar ATPase-inhibitor bafilomycin A (20). ACKR1 levels after blood removal declined similarly with or without bafilomycin A (**Figures 6D–F**), despite the expected inhibition of both lysosomal acidification and autophagy by the drug. Together these data indicate that ACKR1 is not degraded through the proteasome, lysosomes or via autophagic pathways.

Matrix metalloproteinases (MMPs) are enzymes anchored to the cell membrane or secreted into the extracellular matrix and function in the extracellular environment of cells to degrade both matrix and non-matrix proteins. Since ACKR1 is a seven-transmembrane protein on the cell surface with multiple predicted cleavage sites at the extracellular domain, we wanted to investigate whether matrix metalloproteinases (MMPs) are involved in its cleavage and subsequent clearance. Accordingly, we treated cells with Marimastat, a general inhibitor of MMPs, but observed no significant retention of ACKR1 protein upon blood removal (**Figures 6G, H**); this is despite our own data showing that the drug prevents cleavage of the tight junction protein claudin-5 at the same concentration and in the same cells (21).

### ACKR1 is removed from lung microvascular endothelial cells by extracellular vesicles

**Figure 7:**
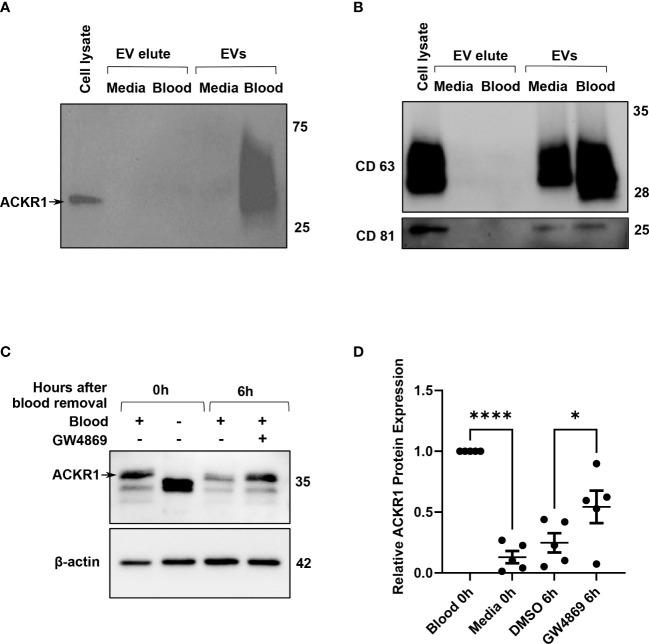
ACKR1 is secreted in extracellular vesicles after removal of blood. HPMECs were incubated with blood or media for 24 hours and subsequently washed and incubated with serum-free media for 6 hours. **(A, B)** Extracellular vesicles (EVs) in the supernatant at 6 hours after blood removal were isolated by size-exclusion chromatography; ACKR1 (using the NBP2-75197 antibody) and EV markers CD63 and CD81 were detected by immunoblotting. **(C)** Cells were treated with the EV inhibitor GW4869 (2 μM) after blood removal for 6 hours. Blot shows whole cell lysate at 0 and 6 hours after blood removal with or without the inhibitor. **(D)** Quantification of ACKR1 levels in cell lysates (****p<0.0001; *p<0.05).

Finally, we considered the possibility that ACKR1 was being secreted by the endothelial cells after blood removal. We were unable to detect the protein in cell culture supernatants by immunoblotting or by ELISA (**Supplemental Figure 2** and data not shown) despite its loss from cell lysates. Hypothesizing that ACKR1 secretion might occur in extracellular vesicles (EV), we next enriched EVs from the cell culture supernatant by size-exclusion chromatography (22). Successful EV isolation was confirmed by detection of CD63 and CD81 (23). ACKR1 was detected in EVs isolated from endothelial cells within 6 hours after the removal of blood and (as expected) was absent in EVs from cells never exposed to blood (**Figures 7A, B**). Formation of EVs requires the sphingolipid ceramide and inhibition of ceramide formation by blocking neutral sphingomyelinase 2 activity has been shown to blunt EV release (24). We observed that inhibition of EV secretion by the neutral sphingomyelinase 2 inhibitor GW4869 (24, 25) significantly attenuated the loss of ACKR1 from endothelial cells (**Figures 7C, D**). These data indicate that upon removal of blood, secretion of ACKR1 in EVs accounts at least in part for its rapid loss from cultured lung microvascular endothelial cells.

### ACKR1 induced by whole blood does not initiate intracellular signalling in response to IL-8 or CXCL1

**Figure 8:**
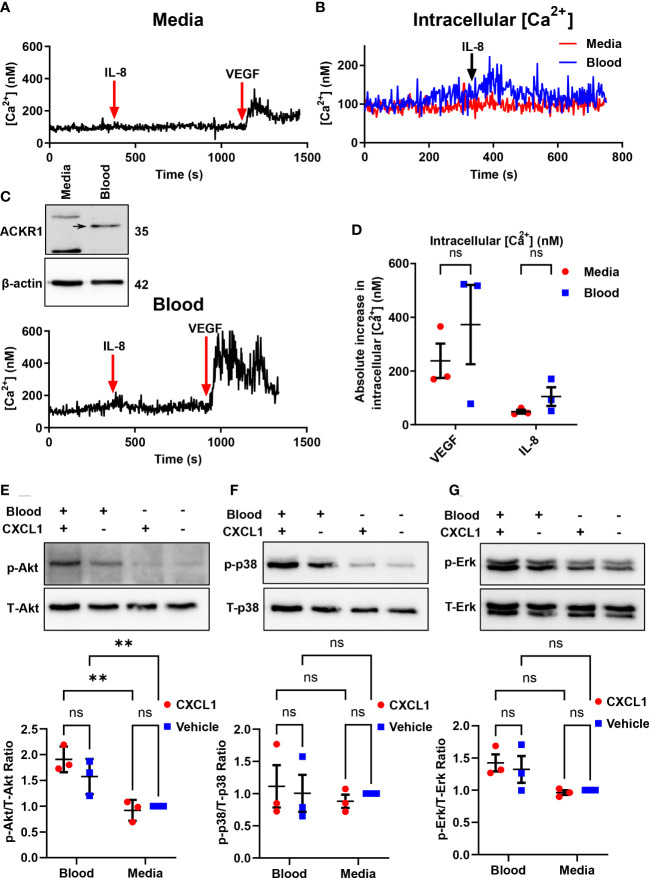
Endothelial ACKR1 does not signal upon cytokine stimulation. HPMECs were incubated with blood for 24 hours followed by Fura2-AM loading for calcium measurements. **(A, C)** are representative measurements of intracellular Ca^2+^ concentration in cells treated with IL-8 (100 ng/ml) for 10 minutes followed by VEGF (40 ng/ml) as a positive control. Inset in C) confirms induction of ACKR1 by blood. **(B)** A merged graph of **(A, C)** showing the calcium concentration upon IL-8 treatment. **(D)** Quantification of the change in intracellular Ca^2+^ concentration upon stimulation with IL-8 or VEGF for each replicate. **(E)** ACKR1 was induced by blood on HPMECs followed by treatment for 1 hour with CXCL1 (4 ng/ml). Protein levels of phospho-Akt (p-Akt) and total Akt (T-Akt) were detected by immunoblotting. **(F)** In the same experiment, protein levels of phospho-p38 and total-p38 and **(G)** phospho-Erk and total-Erk were detected by immunoblotting; ** p<0.01. ns means not significant.

ACKR1 is a seven-transmembrane chemokine receptor with a high affinity for both CC and CXC chemokines (11). The receptor is atypical because it lacks the DRYLAIV sequence motif in the second intracellular loop that is necessary for G-protein coupling in order to signal. Furthermore, Neote et al. reported that HEK293 cells transfected with exogenous ACKR1 do not induce calcium mobilization in response to interleukin-8 (IL-8) or to RANTES (11). As these findings required ectopic expression of ACKR1 in non-endothelial cells, we wished to leverage the ability to induce endogenous ACKR1 expression in the microvascular endothelium to test its capacity for signal transduction. Upon induction of ACKR1 protein, lung microvascular endothelial cells were loaded with Fura2-AM and treated with IL-8 with real-time monitoring of intracellular Ca^2+^ concentrations by ratiometric fluorescence (26). While cells incubated with VEGF as a positive control demonstrated a robust increase in intracellular calcium concentrations, there was no significant difference in calcium influx between control and blood-exposed microvascular endothelial cells in response to IL-8 (**Figures 8A–D**). This is despite persistent ACKR1 expression in the cells as measured by immunoblotting immediately after the experiment. Of note, in another study the ERK-1/2 MAPK pathway was shown to be activated by CXCL1 in an ACKR1-dependent manner in airway smooth muscle cells (27). We treated lung microvascular endothelial cells with CXCL1 for 1 hour after ACKR1 induction and measured the levels of phosphorylated (p)-Akt, (total) t-Akt, p-p38, t-p38, p-Erk and t-Erk. Again, we observed no differences in signaling between blood-exposed and control cells upon treatment with CXCL1 (**Figures 8E–G**). Together, our results indicate that the endogenous ACKR1 protein on lung microvascular endothelial cells cannot signal in response to stimulation with IL-8 or CXCL1.

## Discussion

ACKR1 has 4 extracellular domains separated by 7 transmembrane domains (11) and is highly conserved among primates (28). The erythrocyte protein is a target for *Plasmodium vivax* and *knowlesi* (1), causative agents for malaria; it also plays a role in regulating hematopoiesis (29). In addition, ACKR1 is expressed on the endothelium of post-capillary venules (5, 6) where it is up regulated by inflammation (30, 31); it is absent from larger veins and other blood vessels. Its presence on the endothelium has been shown to confer susceptibility to *S. aureus* leukocidins (10).

The physiological importance of both erythrocyte and endothelial ACKR1 has long been debated (32). Homozygous mutation in a GATA motif in the promoter region causes loss of expression on erythrocytes (i.e., the Fy(a-b-) or Duffy-null phenotype) but not on the endothelium (3); this mutation is common, appearing in two-thirds of African-Americans (33). While the loss of the protein on erythrocytes has no known pathological consequences, other mutations causing complete loss of tissue expression (including from the endothelium) are much less common (33). These few individuals who have been described appeared healthy but the infrequency of this genotype could also be consistent with an important role for endothelial ACKR1. However, the rapid disappearance of ACKR1 from endothelial cells when extracted from tissue (7) has meant that studies of its function and regulation have largely relied on its over-expression in non-endothelial cells or on the use of transgenic mice.

Here we report that contact with blood is sufficient to induce *ACKR1* mRNA and protein expression on human primary lung microvascular endothelial cells. The effect requires contact with neutrophils and is attenuated by inhibition of NF-κB. We confirm that endogenous ACKR1 does not signal in response to stimulation with IL-8 or CXCL1. Instead, ACKR1 expression is rapidly cleared from endothelial cells by secretion in extracellular vesicles (EVs) after blood is removed.

Our data address a long-standing question in vascular biology about the absence of endothelial ACKR1 in cultured cells. Numerous endothelial transcripts and proteins are down-regulated when the cells are extracted from tissue (7); analogously, cultured endothelial cells rapidly lose caveolae (8). This phenomenon has been postulated to be due to loss of cues from the *in vivo* environment. In the case of ACKR1, our experiments indicate that physical contact with neutrophils *in vitro* is sufficient to reverse this loss. Whether this represents a general phenomenon for endothelial proteins or is more specific to ACKR1 and potentially other proteins important for innate immunity is the subject of ongoing work in our laboratory.

The restricted expression of endothelial ACKR1 on post-capillary venules coincides with the region of the vasculature where leukocyte emigration occurs (34). A number of reports have suggested that endothelial ACKR1 regulates chemokine signalling and neutrophil transmigration by binding and displaying cytokines at the cell surface (2, 35, 36). ACKR1 expression has also been observed to be enhanced at the site of suppurative pneumonia in patients (31); concordantly, transgenic mice that over-express ACKR1 on the endothelium exhibit greater leukocyte extravasation in response to injection of CXCL1 (2). Endothelial ACKR1 has been postulated to mediate the translocation of chemokines by caveolae from the interstitial space to the luminal surface of blood vessels (35).More recently, it has been suggested that ACKR1 is enriched at cell-cell junctions of the endothelium, where it may serve to bind chemokines and guide the process of leukocyte emigration (36). Mice globally-deficient in ACKR1 were transplanted with *Lyz2-EGFP-ki/DARC^+/+^* bone marrow, endowing the knockout mice with hematopoietic (but not endothelial) ACKR1 expression. In this model, neutrophil emigration to TNF-stimulated tissues was impaired.

If ACKR1 serves to enhance the innate immune response, our experiments now indicate how this effect can be terminated with removal of the inciting stimulus; endothelial ACKR1 is rapidly cleared by its secretion into EVs. Beyond down-regulation of the immune response, whether ACKR1 in EVs has additional functions (e.g. by transmitting inflammatory signalling to distant cells) is unclear and is the subject of ongoing work. In addition to the removal of the protein by EVs, the rapid loss of ACKR1 mRNA suggests additional regulation at the level of transcription. Together, our findings that ACKR1 expression is up-regulated by endothelial-neutrophil contact and then rapidly removed by EVs is consistent with it playing an important role in titrating the local innate immune response. The ability to induce endothelial ACKR1 expression *in vitro* may be a valuable tool to permit further elucidation of its physiological functions.

## Data availability statement

The raw data supporting the conclusions of this article will be made available by the authors, without undue reservation.

## Ethics statement

The studies involving human participants were reviewed and approved by St. Michael’s Hospital Research Ethics Board. The patients/participants provided their written informed consent to participate in this study.

## Author contributions

The authors confirm contribution to the paper as follows: Study conception and design: WL, XG, NK, VT, AK. Data collection: XG, NK, SR, JS, NF, MA. Analysis and interpretation of results: WL, XG, NK, AK, KH, JF, VT. Draft manuscript preparation: WL, XG, NK. All authors reviewed the results and approved the final version of the manuscript. All authors contributed to the article and approved the submitted version.

## Supplementary material

Supplementary Figure 1 Endothelial ACKR1 is induced by blood from different donors and detected by different antibodies. **(A)** Representative immunoblotting for ACKR1 on lung microvascular endothelial cells incubated with media or blood from four different donors for 24 hours. The same 35 kDa band (ACKR1’s predicted molecular weight) was detected by both the Ab58965 polyclonal antibody (no longer available) and NBP2-75197 monoclonal antibody and was selected for quantification throughout the paper. **(B)** Antibody binding sites against ACKR1.

Supplementary Figure 2 ACKR1 was not detected in cell supernatant after removal of blood. HPMECs were incubated with blood or complete media for 24 hours then washed and incubated with media. **(A)** Immunoblot for ACKR1 in cell lysates at various time points after removal of blood. **(B)** Cell supernatant from different time points was tested by immunoblotting for ACKR1 after acetone precipitation.
