# Supplementary material for: Endothelial ACKR1 is induced by neutrophil contact and down-regulated by secretion in extracellular vesicles
Source: Front Immunol. 2023 Apr 21;14:1181016. doi: 10.3389/fimmu.2023.1181016 (PMC10160463; doi:10.3389/fimmu.2023.1181016)
Supplement: Supplementary Figure 1 — Endothelial ACKR1 is induced by blood from different donors and detected by different antibodies. (A) Representative immunoblotting for ACKR1 on lung microvascular endothelial cells incubated with media or blood from four different donors for 24 hours. The same 35 kDa band (ACKR1’s predicted molecular weight) was detected by both the Ab58965 polyclonal antibody (no longer available) and NBP2-75197 monoclonal antibody and was selected for quantification throughout the paper. (B) Antibody binding sites against ACKR1. [file Image_1.pdf]

Supplemental Figure 1.

A

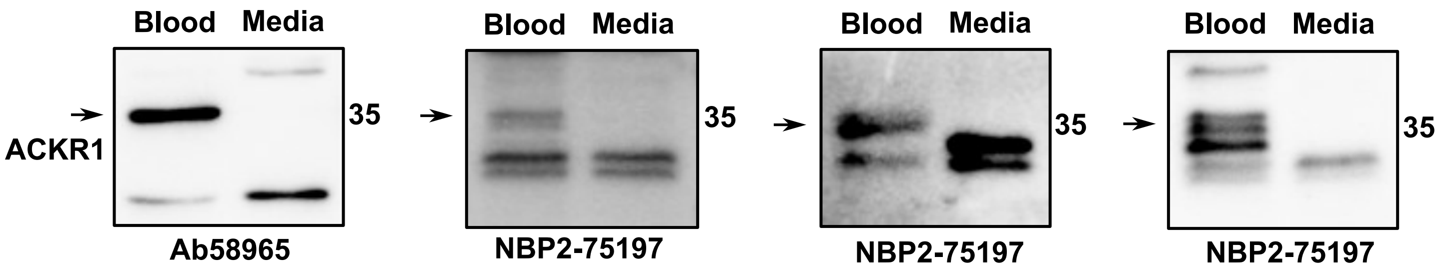

B

ACKR1 antibody binding sites

```
MASSGYVLQA ELSPSTENSS QLDFEDVWNS SYGVNDSFPD GDYGANLEAA APCHSCNLLD      60
                     NBP2-75197 binding site
DSALPFFILT SVLGILASST VLFMLFRPLF RWQLCPGWPV LAQLAVGSAL FSIVVPVLAP     120

GLGSTRSSAL CSLGYCVWYG SAFAQALLLG CHASLGHRLG AGQVPGLTLG LTVGIWGVAA     180

LLTLPVTLAS GASGGLCTLI YSTELKALQA THTVACLAIF VLLPLGLFGA KGLKKALGMG     240

PGPWMNILWA WFIFWWPHGV VLGLDFLVRS KLLLLSTCLA QQALDLLLNL AEALAILHCV     300

ATPLLLALFC HQATRTLLPS LPLPEGWSSH LDTLGSKS      338
            Immunogen for Ab58965
```
