# Supplementary figures and images for: Endothelial ACKR1 is induced by neutrophil contact and down-regulated by secretion in extracellular vesicles
Source: Front Immunol. 2023 Apr 21;14:1181016. doi: 10.3389/fimmu.2023.1181016 (PMC10160463; doi:10.3389/fimmu.2023.1181016)

Supplemental Figure 2.

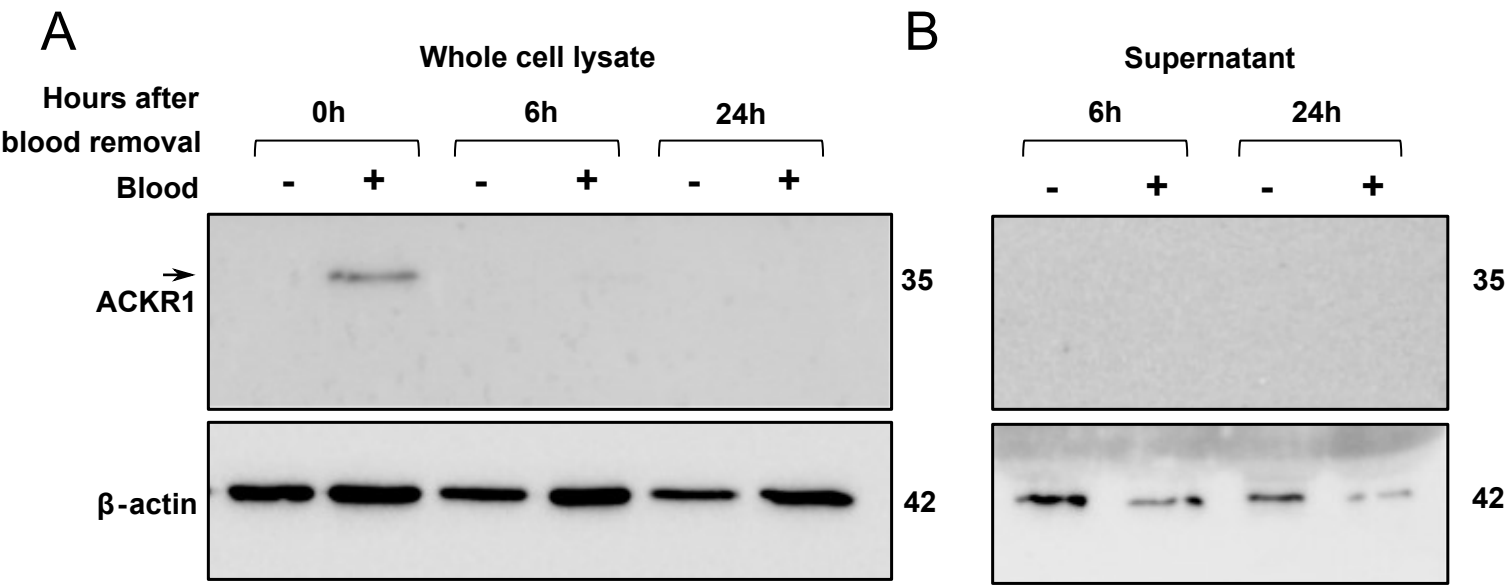

Supplement: Supplementary Figure 2 — ACKR1 was not detected in cell supernatant after removal of blood. HPMECs were incubated with blood or complete media for 24 hours then washed and incubated with media. (A) Immunoblot for ACKR1 in cell lysates at various time points after removal of blood. (B) Cell supernatant from different time points was tested by immunoblotting for ACKR1 after acetone precipitation. [file Image_2.pdf]
